# Structure and development of the complex helmet of treehoppers (Insecta: Hemiptera: Membracidae)

**DOI:** 10.1186/s40851-020-00155-7

**Published:** 2020-02-24

**Authors:** Haruhiko Adachi, Keisuke Matsuda, Kenji Nishida, Paul Hanson, Shigeru Kondo, Hiroki Gotoh

**Affiliations:** 1 Graduate School of Frontier Bioscience, Osaka University, Suita, Osaka Japan; 2 Collaborator, Museo de Zoología, Universidad de Costa Rica and Estación Biológica Monteverde, Monteverde, Puntarenas, Costa Rica; 3 Escuela de Biología, Universidad de Costa Rica, San Pedro de Montes de Oca, San José, Costa Rica; 4 Faculty of Environmental Earth Sciences, Hokkaido University, Sapporo, Hokkaido Japan; 5 Ecological Genetics Laboratory, Department of Genomics and Evolutionary Biology, National Institute of Genetics, Mishima, Shizuoka 411-8540 Japan

**Keywords:** Morphogenesis, 3D structure, Treehopper, Membracidae, Pronotum, Micro-CT

## Abstract

Some insects possess complex three-dimensional (3D) structures that develop under the old cuticle prior to the last imaginal molt. Adult treehoppers (Insecta: Hemiptera: Auchenorrhyncha: Membracidae) have one such complex 3D structure, known as a helmet, on their dorsal side. The adult helmet likely forms inside the nymphal pronotum during the final instar nymphal stage.

Previous morphological studies have reported that the adult helmet is a large, bi-layered, plywood-like structure, whereas the nymphal pronotum is a monolayer, sheath-like structure. The adult helmet is much larger than nymphal helmet. Thus, the emergence of the adult helmet involves two structural transitions: a transition from a monolayer, sheath-like pronotum to a bi-layer, plywood-like helmet, and a transition in size from small to large. However, when, how, and in what order these transitions occur within the nymphal cuticle is largely unknown.

To determine how adult helmet development occurs under the nymphal cuticle, in the present study we describe the morphology of the final adult helmet and investigate developmental trajectories of the helmet during the final instar nymphal stage. We used micro-CT, scanning electron microscope and paraffin sections for morphological observations, and used *Antianthe expansa* as a model species.

We found that the structural transition (from monolayer, sheath-like structure to bi-layer, roof-like structure) occurs through the formation of a “miniature” of the adult helmet during the middle stage of development and that subsequently, extensive folding and furrows form, which account for the increase in size. We suggest that the making of a “miniature” is the key developmental step for the formation of various 3D structures of treehopper helmets.

## Background

Arthropods are covered with a hard exoskeleton that physically and chemically protects the body [1–3]. However, due to its limited elasticity, the exoskeleton prevents continuous growth; most arthropods species therefore grow via shedding their hard cuticle, a phenomenon known as molting [2–4]. Before molting, a new exoskeleton forms under the old exoskeleton. A newly developed exoskeleton has extensive furrows that enable it to expand into complex, three-dimensional (3D) structures when the furrows are extended [3]. Recently, Matsuda et al. (2017) experimentally and via computer simulation demonstrated that the later extension process is a simple physical transition that involves no cytological activities, such as cell division, migration, or apoptosis [5]. Thus, the final structure is uniquely determined when the primordial morphogenesis has been completed. Considering the diversity of arthropod exoskeleton morphology, this “morphogenesis via folding and extension” may be able to form various final 3D shapes. However, the developmental logistics of such primordia with specific furrows and coding of various 3D structures remains to be understood.

**Fig. 1:**
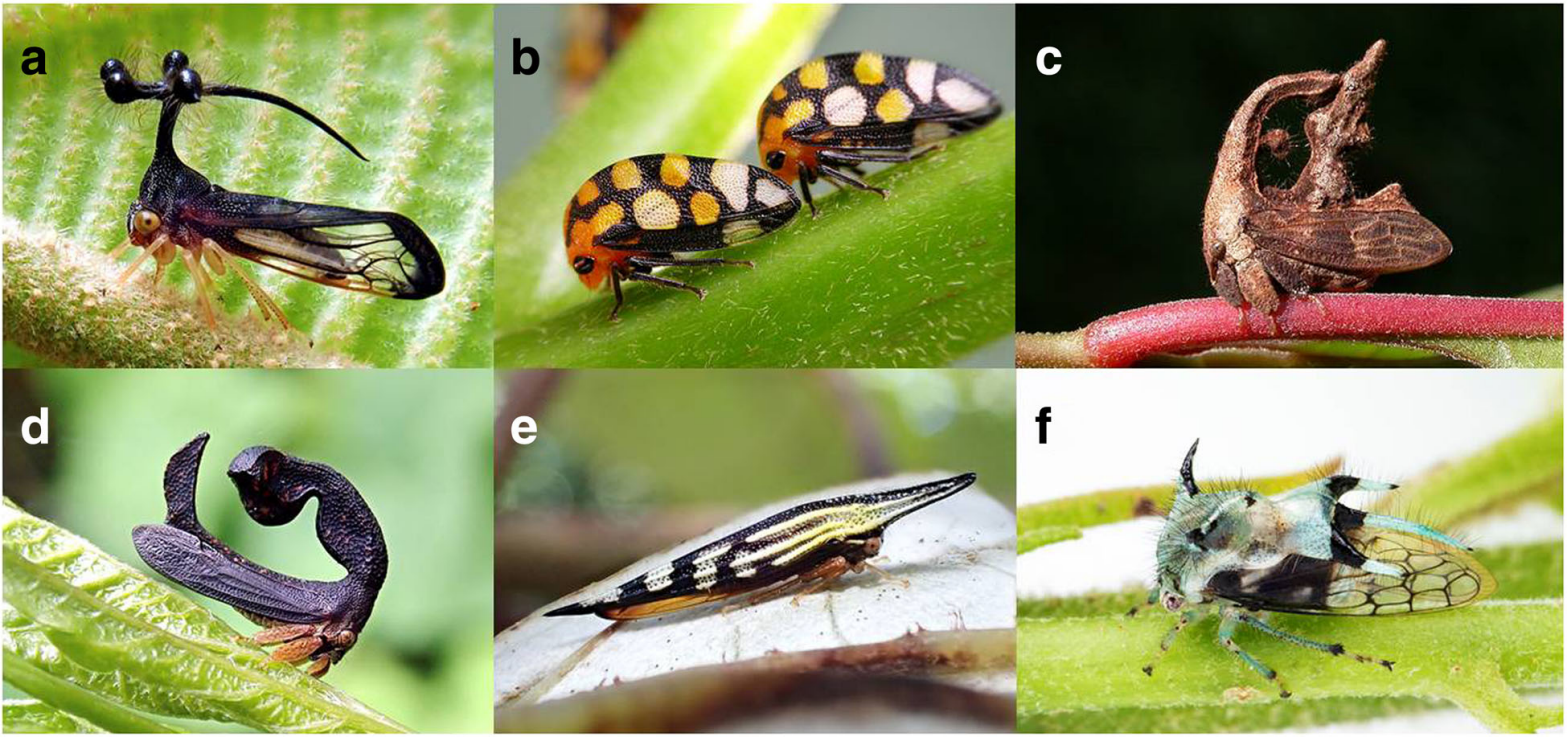
Various 3D morphologies of treehopper helmets. The morphology and color of treehopper helmets show a wide diversity. All species shown here occur in Costa Rica: **a***Bocydium mae.***b***Addipe zebrina,***c***Cladonota biclavata,***d***Cladonota* sp., **e***Polyglypta* sp.*,***f***Poppea* sp


One of the most extreme examples of 3D structures formed by “folding and extension” is the helmet (an enlarged pronotum) of treehoppers (Insecta: Hemiptera: Auchenorrhyncha: Membracidae) (Fig. 1a-f). The 3D shapes of treehopper helmets are extremely diverse, including branched TV antenna-like (e.g. *Bocydium* Fig. 1a), roof-like (e.g. *Adippe* Fig. 1b), crescent or more complex C shape (e.g. *Cladonota* Fig. 1c-d), elongated cylindrical (e.g. *Polyglypta* Fig. 1e), spiny to forked (e.g. *Poppea* Fig. 1f) or ant-mimicking [6–8] shapes. Such complex 3D shapes appear at the time of molting from a final instar nymph to an adult [9, 10]. Over a short time period both the size and shape of the helmet are dramatically transformed at molting. Considering this “transformation” (Supplementary Movies 1, 2, 3, 4 and 5), i.e. emergence of the adult helmet, it is likely that the specific folding and furrow patterns are already developed on the helmet primordium located below the cuticle of the last instar nymph.

**Additional file 1: Movie S1.** Eclosion of *Antianthe expansa*, Original movie (approx. 35 min) was compressed into 21 s.



**Additional file 2: Movie S2.** Eclosion of *Bocydium* sp. Original movie (approx. 30 min) was compressed into 27 s.



**Additional file 3: Movie S3.** Eclosion of *Cladonota* sp. Original movie (approx. 40 min) was compressed into 25 s.



**Additional file 4: Movie S4.** Eclosion of *Umbonia* sp. Original movie (approx. 60 min) was compressed into 39 s.



**Additional file 5: Movie S5.** Eclosion of *Poppea capricornis*. Original movie (approx. 30 min) was compressed into 42 s.


Previous morphological studies have indicated that the adult helmet is a bi-layer, plywood-like structure while the nymphal pronotum is a monolayer, sheath-like structure [9]. Thus, the emergence of the adult helmet includes two structural transitions, that is, 1) transition from a monolayer, sheath-like pronotum to a bi-layer, plywood-like helmet and, 2) transition in size from a small helmet to a large helmet. When, how, and in what order do these two transitions occur inside the nymphal cuticle?

Except for a few molecular studies focusing on wing developmental genes [10] and on transcriptomic profile [11], few studies of treehopper helmet morphogenesis have been reported; histological and anatomical descriptions are particularly scarce. In the present study, we used a treehopper species, *Antianthe expansa* (Germar, 1835) (Smiliinae: Smiliini) which has a roof-shaped helmet, to reveal the two structural transitions and demonstrate developmental trajectories and helmet structure during the final instar nymph by using micro-CT, scanning electron microscopy (SEM), and paraffin sections.

## Results and discussion

### Structure of adult helmet

**Fig. 2:**
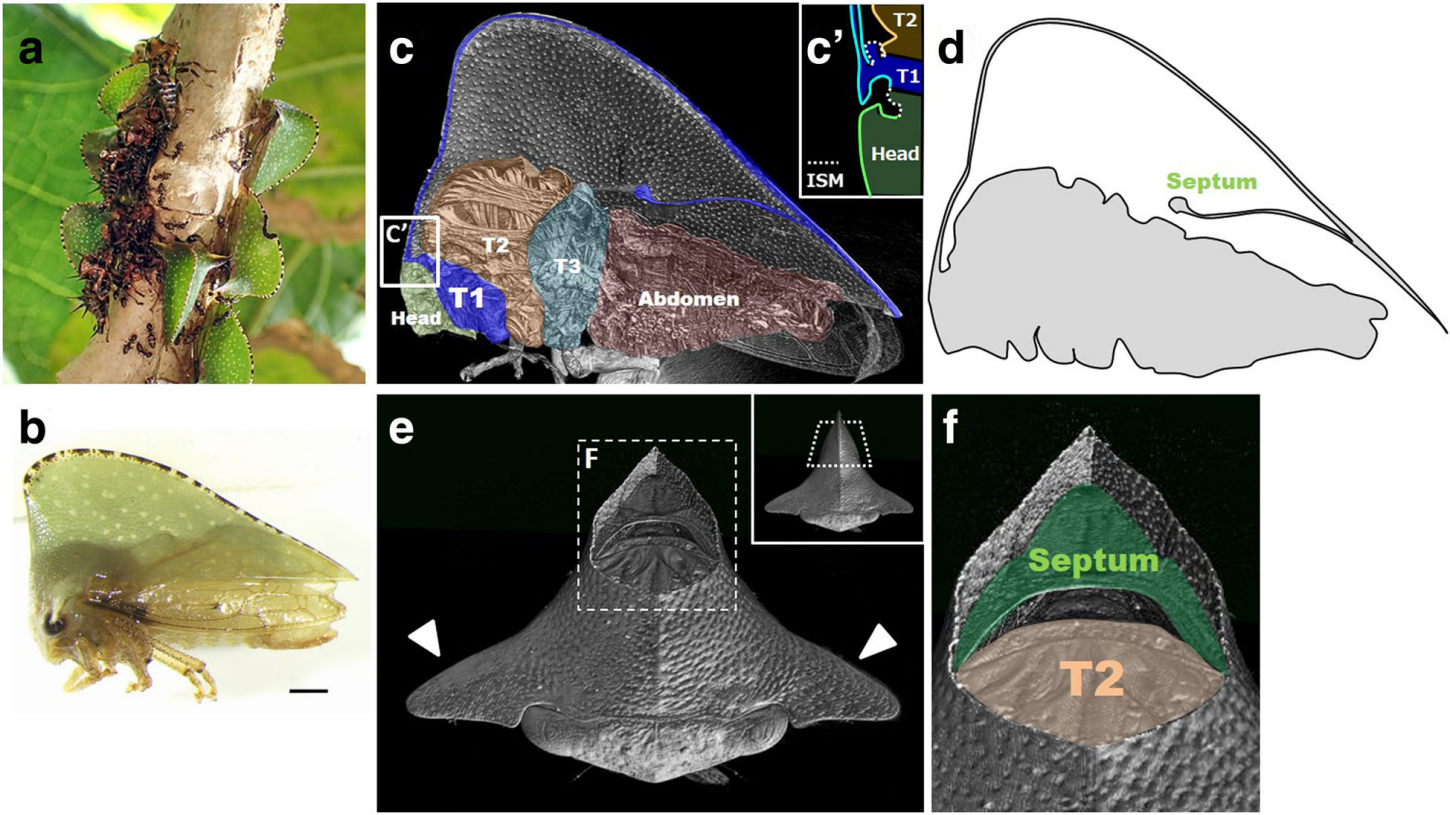
Adult morphology of focal species, *Antianthe expansa.***a** Adults and nymphs of *Antianthe expansa* in the field. Adults and various nymphal instars are present on the same branch and are often associated with ants. **b** Side view of adult. Scale bar indicates 1 mm. **c** Virtual sagittal section of adult via micro-CT scan imaging. Head, prothorax (T1), mesothorax (T2), metathorax (T3) and abdomen are indicated in different colors. Connection point of head, helmet (dorsal prothorax), and mesothorax is magnified in C’. ISM:  intersegmental membrane. **d** Diagram of cross section of C. It can be clearly seen that the helmet is a thin structure and connects only at the most anterior region. **e** Frontal view of adult via micro-CT imaging. The dorsalmost region of the helmet was graphically excised to show the inner side of the helmet. White arrows indicate a pair of lateral projections (humeral horns). **f** Magnified image of dashed window in E. Highlighted in green is the septum and in orange is dorsal part of the mesothorax (T2)

The helmet of the adult *Antianthe expansa* covers most of the dorsum of the body [7] (Fig. 2a-d) and has a pair of lateral projections (humeral horns) at its anterior end (Fig. 2e). The helmet is connected to the main body only at its anterior margin (Fig. 2c, C’), i.e., covering over the mesothorax, metathorax, and abdomen with no other connections (Fig. 2c, d). Thus, the venter of the helmet is actually external to the body.

**Fig. 3:**
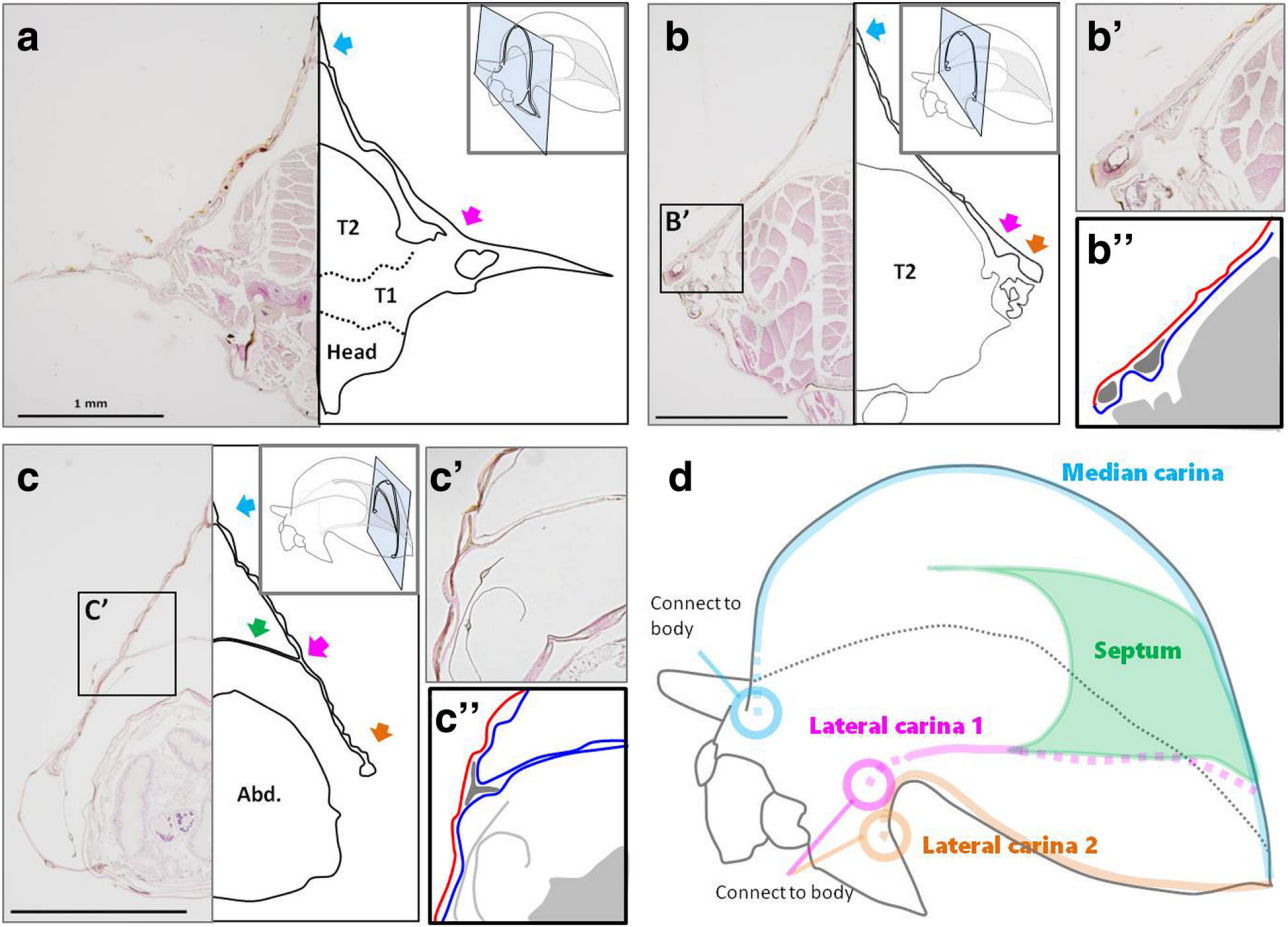
Structure of adult helmet of *Antianthe expansa*. **a**-**c** Frontal section of adult *Antianthe expansa*. **a** Frontal section at lateral projection. A connection between lateral carina 1 (pink arrow) and the body is clearly recognizable. There is a tubular structure at the top of the helmet known as the median carina (blue arrow). **b** Frontal section at middle mesothorax. Note that the helmet and body are not connected. There are two tubular lateral carinae on each side of the helmet (pink and orange arrow). B′) Magnified image of lateral carinae which have a thicker cuticle. Also, it can be clearly seen that the helmet is double layered. B″) Schematic drawing of B′. Red and blue line indicate upper and lower layer, respectively. Dark gray indicates inner space of lateral carinae. **c** Frontal section at abdomen. The septum (green arrow) bridges both sides of the helmet. C′) Magnified image of connected area of septum. The septum is composed of two thin layers of cuticle, so that the septum is also double layered. C″) Schematic drawing of C. Red and blue line indicate upper and lower layer, respectively. Both of the two layers comprising the septum are from the lower layer. **d** Schematic summary of adult helmet structure

In the posterior half of the helmet, there is a wall-like structure that bridges the left and right sides (Fig. 2c-f). This structure is known as the septum, and was first named by Buckton and Poulton in 1903 [9, 12]. Detailed observation of the helmet using paraffin sections indicated that the helmet is composed of a bi-layered cuticle (Fig. 3B’, B″, C′, C″). The space between the two layers is connected to the body. Some unstructured tissues and cells were observed between these layers (Fig. 3B’, B″, C′, C″). The apical area (median carina) (light blue arrows in Fig. 3a-c) and the bottom of the helmet, beginning at the base of humeral horns and extending posteriorly (orange arrows in Fig. 3a-c), are enlarged (with an interlayer space) to form tubular structures. These tubular structures have also been described as median carina and lateral carinae in a previous study [9]. The median carina connects to the body at the prothorax, just posterior to the head, and the lateral carinae connect at the lateral projection (Fig. 3d). Since the carinae have a thicker cuticle than the other areas of the helmet (Fig. 3b, B’), they are likely to function as a framework for the helmet. The carinae also function as pathways for body fluid that is pumped at the imaginal molt in order to extend the densely folded helmet primordium. The septum is also composed of two layers, both of which are connected to the lower layer (also known as the ventral lobe [9]) of the helmet (Fig. 3C’, C″). In other words, the lower layer is folded inside with the inner folded layers attached to each other to form the septum (Fig. 3c). These structures (bi-layer helmet, median carina, lateral carinae, and septum) were observed in a previously studied species, *Stictocephala bisonia*, which also has roof-like helmet [9]. One difference between *Antianthe* and *Stictocephala* is that the *Antianthe* has two pairs of lateral carinae (Fig. 3b-d) while the *Stictocephala* has just one pair.

### Structure of nymphal pronotum

**Fig. 4:**
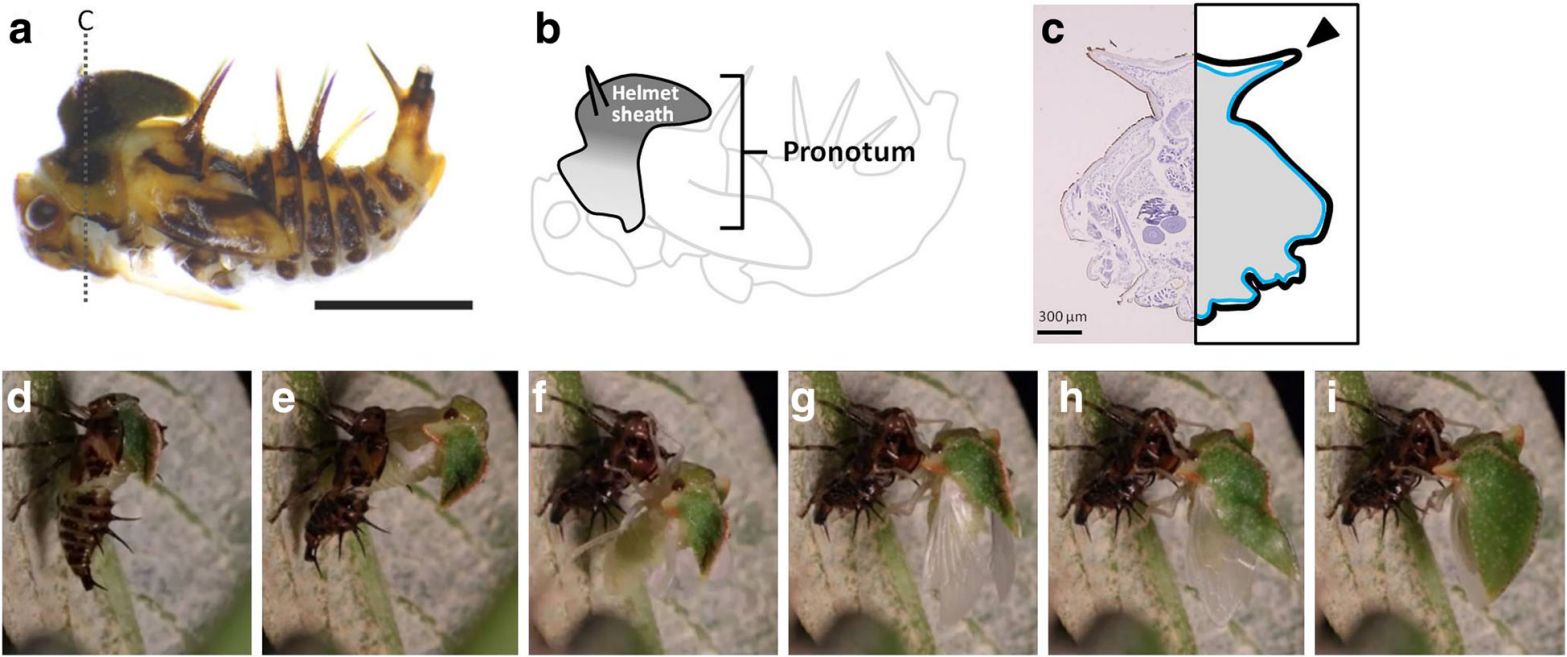
Structure of nymphal pronotum of *Antianthe expansa*. **a** Side view of last instar nymph of *Antianthe expansa* (legs were removed). Scale bar indicates 2 mm. Sectioned region in C is indicated as a dashed line. **b** Schematic summary of pronotum and helmet sheath of final instar nymph. We call the dorsal projected region of the nymphal pronotum a “helmet sheath” indicated in dark gray. There is no clear border between the helmet sheath and other parts of the pronotum. **b** Frontal section of nymph. Note that the helmet sheath is monolayer (the epithelium is indicated by a blue line) and the inside of the helmet sheath is filled with tissue. **c**-**h** Time-lapse images of eclosion of an adult *Antianthe expansa*. Extremely folded structures (dense furrows) were extended to form the final 3D helmet structure. This transformation occurs in approximately 35 min, which indicates that cytological activities (e.g. cell division and migration, etc.) cannot play important roles in this extension

In the last nymphal instar the dorsal area of the pronotum extends to the posterior part of mesothorax forming a “helmet sheath” that contains part of the folded adult helmet (Fig. 4a, b). This helmet sheath is a single layer and the inner surface of the sheath is connected to the body (Fig. 4b). As Stegmann (1998) mentioned in his study [9], the nymphal pronotum is a large, single-layered outgrowth of integument connecting with the body cavity. On the helmet sheath, there is a pair of thorn-like horns projecting laterally (Fig. 4a, b, c arrow); however, these are missing in the adult stage. At the imaginal molt, the developed adult helmet formed inside the helmet sheath extends into a large roof-like helmet (Fig. 4d-i). This extension of the folded helmet likely occurs by pumping hemolymph (body fluid) into the median and lateral carinae, similar to the extension of wings by pumping hemolymph into wing veins. This expansion process occurs during a short time period. Also, the less-elastic cuticle layers have already formed on the surface of helmet at this stage. Therefore, cytological activities, such as cell division, are not likely to be involved in this transition, similar to the transformation of a densely folded horn primordium into a long bifurcated horn seen in rhinoceros beetles [5]. Thus, there are two transitions: 1) structural transition from a monolayer sheath-like structure into bi-layer plywood-like structure, and 2) an increase in the size of the helmet. Preparations for these transitions are complete before the final molt. To investigate how these two transitions are achieved inside the nymphal helmet sheath, we next analyzed the development of the helmet during the last nymphal stage.

### Morphogenetic processes of helmet during nymphal stage

**Fig. 5:**
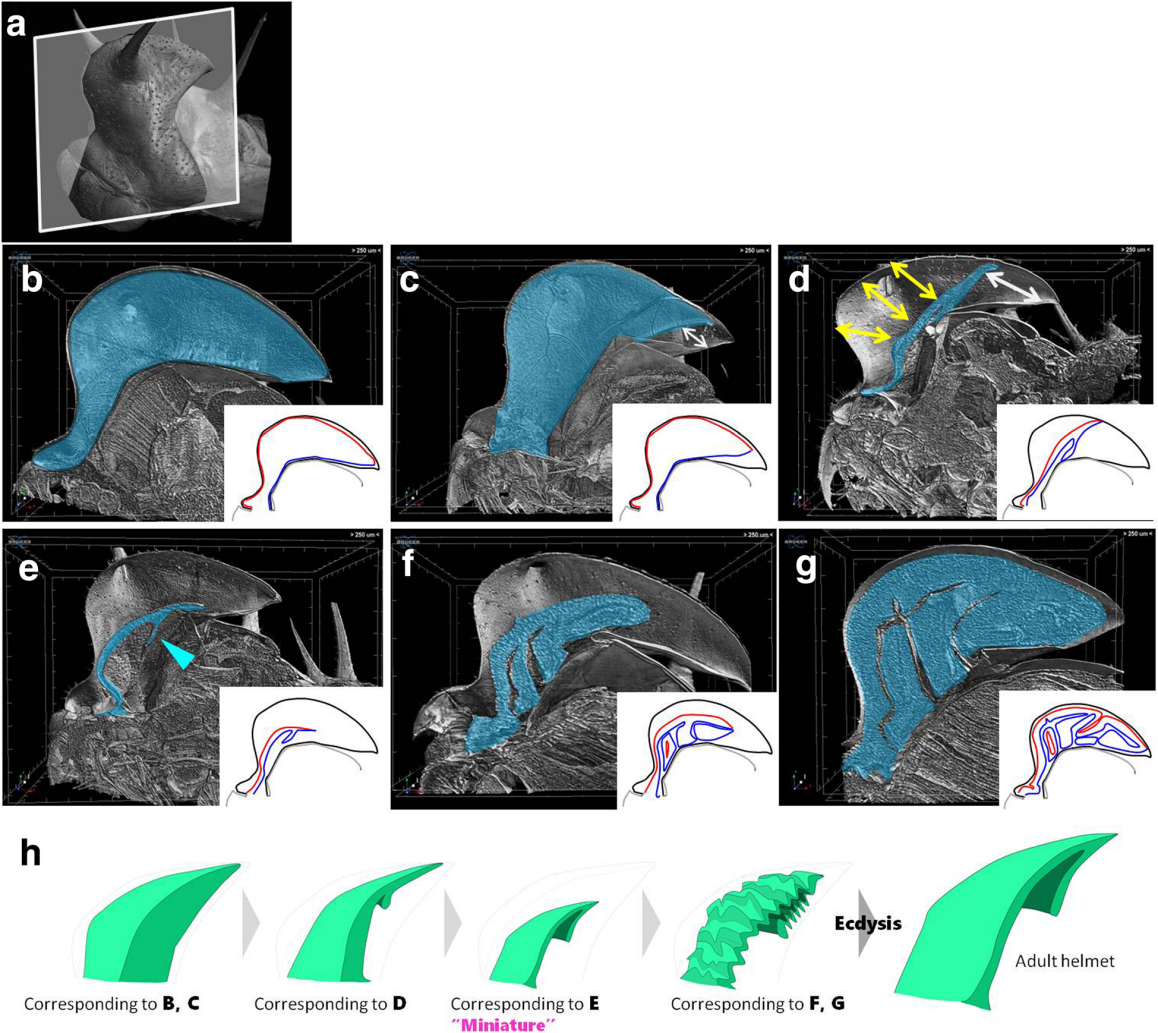
Helmet morphogenesis inside the nymphal helmet sheath in *Antianthe expansa.***a** Surface view of last instar nymph of *Antianthe expansa* via micro CT scan. White window indicates sectioned region in B to G. **b**-**g** Sagittal section of last instar nymph. The developing adult helmet primordium is highlighted in blue. Schematic diagrams of developing helmet are shown in small windows in each panel. Black lines, red lines and blue lines indicate nymphal cuticle, upper layer and lower layer, respectively. **b** At an early stage of the last instar nymph (before apolysis), the helmet was a monolayer, sac-like structure. **c** Once apolysis started, the helmet epithelium detached from the nymphal cuticle. First, the lower layer of most posterior region detached, indicated by a white arrow. **d** Both upper and lower layer detached from the nymphal cuticle (yellow arrows and white arrow) and came together to form a thin, double-layered plate-like structure. **e**Shrunken miniature of adult helmet has formed. At this stage a bi-layer septum also formed (blue arrowhead). **f** Miniature grows, probably by cell proliferation. During this stage both macro and micro furrows are developed. **g** Fully developed helmet primordium which fills the entire space of the helmet sheath. The bi-layer epithelial sheet is highly complex in three dimensions, so that it is difficult to understand its structure from a single section. **h** Schematic diagram of helmet development during the last instar nymph

In order to investigate the developmental trajectory of the helmet, we observed the inner developing helmet structure of the final instar nymph by using a micro-CT scan (Fig. 5). As we could not keep living treehoppers in the laboratory due to permission restrictions, we collected dozens of final instar nymphs in the field and immediately fixed them. The chronological order was reconstructed based on the development of their wings and flight muscles. In the young final instar, i.e. prior to adult helmet morphogenesis, the epithelia of the pronotum including the helmet sheath were a monolayer covered by nymphal cuticle (Fig. 5b). Adult helmet morphogenesis begins with apolysis (detachment of the epithelial sheet from old cuticle) of the helmet epithelium. Apolysis likely occurs at the most posterior part (Fig. 5c), where the lower layer first detaches from the nymphal cuticle (Fig. 5c, white arrow). Along with the apolysis process, the anterior upper layer also detached from the cuticle (Fig. 5d, yellow arrows). At this stage, the helmet epithelium appears to degenerate like a deflated balloon, that is, the upper and lower layer come together and the internal space of the developing helmet mostly disappears (Fig. 5d). This is a remarkable transition from a single to a double layer (Fig. 5d). The entire newly formed helmet then shrank and apolysis was complete (Fig. 5e). After this stage, the helmet grew larger (Fig. 5f) and finally filled the entire space of the helmet sheath and other areas of the pronotum (Fig. 5g). As the helmet grew, many folded structures were also formed (Fig. 5f, g).

**Fig. 6:**
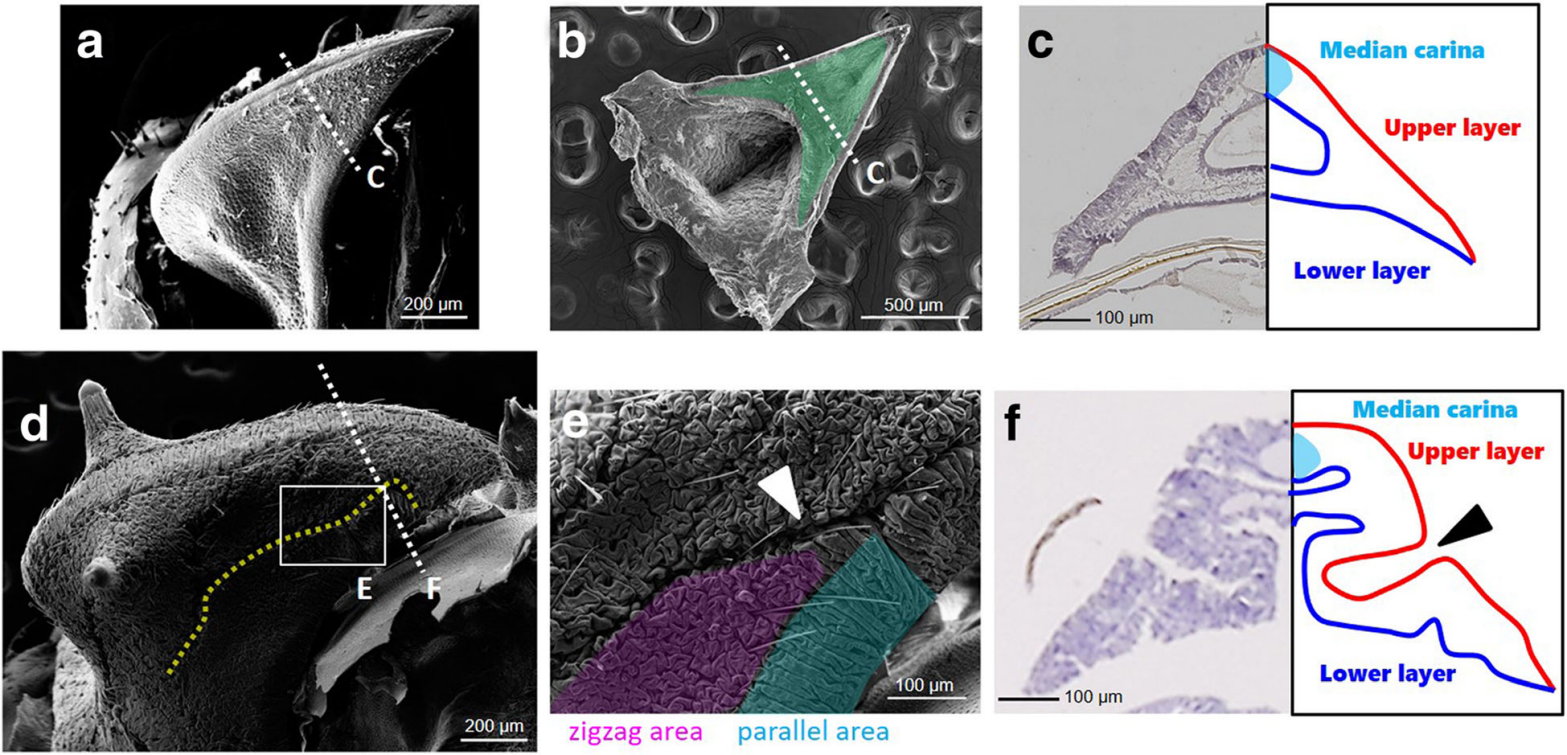
Observation of developing helmet via SEM and paraffin section. **a** Whole morphology of developing miniature helmet by scanning electronic microscope (SEM) after removing outer nymphal cuticles. **b** Ventral view of miniature helmet by SEM. Septum is highlighted in green. **c** Paraffin section of miniature helmet. Upper (red line) and lower (blue line) layers can be recognized. Also, the median carina has already formed at top of the helmet, indicated as light blue. **d** Whole morphology of fully developed helmet by SEM after removing outer nymphal cuticles. **e** Magnified view of surface of the helmet. There are dense micro furrows and deep macro furrow (white arrowhead). Some areas have zigzag micro furrows (highlighted in pink) and some have parallel regular furrows (highlighted in blue). The micro furrows determine the rate and direction of expansion when extended. **f** Paraffin section of fully developed folded helmet. A deep macro furrow was made by bending the bi-layer cell sheet (black arrowhead)

Next, we observed the outer morphology and topological structures of the developing helmet after shrinking (corresponding to Fig. 5e) and the fully developed helmet (corresponding to Fig. 5g) by using scanning electron microscopy (SEM) and paraffin sections. We found that the shape of the developing helmet after shrinking was very similar to that of the adult helmet, although the total helmet size was still much smaller at this stage (Fig. 6a). From the ventral side, the septal structure can also be observed (green highlighted area in Fig. 6b). By observing a cross-section of the helmet, it is apparent that the lower and upper layers have become attached to form bi-layer structure (Fig. 6c). In this paraffin section the median carina and bi-layer septum were also clearly recognized (Fig. 6c). Thus, most of the characteristic components of the adult helmet structure (bi-layer roof shape, septum and median carina) have already appeared by this stage, so this developing helmet can be referred to as a “miniature” of the adult helmet. In other words, the structural transition from a single-layered, sheath-like structure to double-layered roof-shaped structure, one of the two transitions mentioned above, is nearly complete at this stage.

After this stage the miniature helmet grows larger to its final folded structure. As the helmet grows many folded furrows are formed on its surface (Fig. 6d, e, f). This growth with furrow formation is likely responsible for the size transition from a small nymphal helmet sheath to a large adult one. There are at least two kinds of furrows. The first type consists of deep furrows formed by bending the bi-layer epithelial sheet (hereafter macro furrow). The most apparent macro furrow runs along the anterior posterior axis on both sides of the helmet (indicated by a yellow dashed line in Fig. 6d and by arrowheads in Fig. 6e and f). The second type consists of superficial dense furrows seen in most of the surface of the helmet (hereafter micro furrow). Most of these micro furrows are irregular “zigzag” in form (highlighted in pink in Fig. 6e) which enable the helmet to become broadened in every direction. In some specific areas, such as the surface of the median and lateral carina, the micro furrows are regular and parallel (highlighted in blue in Fig. 6e), which enable the tubular structures to expand in one direction. Both macro and micro furrows might be responsible for the enlargement of the helmet and determine the rate and direction of expansion.

Although the cytological contribution to helmet shrinking and its re-growth with folding is still unknown, we suggest that various cell activities (proliferation, shape change, migration and cell death) are involved in those processes. In particular, extensive cell proliferation is highly likely to be involved in the growth of the miniature helmet.

In conclusion, helmet development of *Antianthe expansa* occurs as follows. A sheath-like, monolayer epithelial sheet undergoes apolysis and shrinks into a miniature helmet by the lower and upper layer coming together (structural transition from monolayer to bi-layer). The miniature helmet then grows larger while forming both macro and micro furrows (size transition from small to large) (Fig. 5h).

### Developmental similarities between helmet and wing

**Fig. 7:**
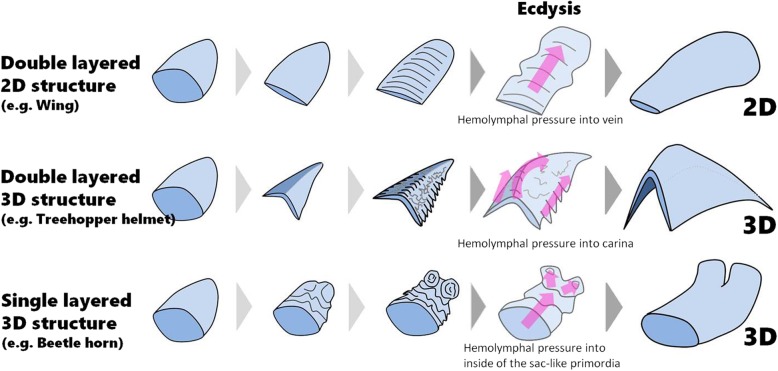
Schematic diagram of 2D and 3D morphogenesis of insect adult structures via “folding and extension”. All structures essentially started from a sac-like monolayer structure, but the final structures differ (2D or 3D, monolayer or double layered). In a bi-layer 2D structure like an insect wing, internal space disappears during development by connecting the upper and lower layers. Then, flat bi-layer primordia form relatively simple parallel furrows which enable primordia to enlarge in a single direction. In a bi-layer 3D structure like the treehopper helmet, upper and lower layers stick together to form a bi-layer sheet. In contrast to the wing, this bi-layer structure has a 3D shape. The macro and micro furrows determine the expansion rate in multiple direction to form its 3D shape. In a monolayer 3D structure, the inner space of the primordium does not disappear during development. Furrows are formed in the surface of the primordium by bending a single layer of epithelium and are extended by hemolymphal pressure at molting

As we have shown, the treehopper develops densely folded primordia before the molt and extends them at molting, thereby forming a complex 3D structure. This developmental pattern is also seen in the development of the rhinoceros beetle horn [5] (Fig. 7). However, there are critical differences between the treehopper helmet and the beetle horn. In the beetle horn, the primordium is a single layered, sac-like structure and is extended by body fluid pressure filling the inside of the sac (Fig. 7). On the other hand, the treehopper helmet is a double layered, plywood-like structure and is extended by body fluid pressure through the tubular carinae (Fig. 7).

In its transformation from a monolayer to a bi-layer structure, the treehopper helmet shows similarity with wing development (Fig. 7). In hemimetabolous insects, the wing develops inside a sac-like wing bud as a monolayer structure, but transforms into a double layered, flat 2D structure during development [2] (Fig. 7). Initially, the bi-layer wing is a small miniature of the adult wing (Fig. 7), but subsequent cell proliferation enables the wing to grow by forming dense furrows via bending the bi-layer sheet (Fig. 7). Even in wing development in the highly-derived *Drosophila*, dorsal and ventral epithelia come together to form a double layered, flat miniature wing during development [13]. Moreover, the furrows are extended by pumping hemolymph through tubular structures in both treehopper helmet (carina) and wing (vein) (Fig. 7). Although anatomical homology between helmet and wing has not been supported [14, 15], recent molecular developmental studies suggest that the wing and helmet share molecular networks. That is, important wing developmental genes expressed in developing helmets [10] and the transcriptomic profile of developing helmets show more similarity to the developing wing than to other body regions (e.g. legs and tergites) [11]. Developmental similarity between helmet and wing shown in this study also supports this idea.

## Conclusion

In this study, we observed the morphogenesis processes of the complex 3D morphology of the treehopper helmet. From the observed developmental pattern, the 3D helmet shape of treehoppers likely forms as follows, 1) the miniature helmet with characteristic adult helmet structures (e.g. bi-layer structure with septum) is formed, 2) the miniature helmet increases in size while forming macro- and micro-furrows, and finally, 3) the furrows are extended at molting to form the 3D helmet morphology. The mechanism for making a miniature of the helmet and area-specific furrow formation should be investigated in future studies in order to better understand one of the most spectacular 3D morphologies seen in the nature.

## Materials and methods

### Insect collection

Treehopper samples, nymphs and adults were collected at the Universidad de Costa Rica, San Pedro de Montes de Oca, San José, Costa Rica in November of 2016 and 2017. Sampled insects were immediately fixed in formalin and 95% EtOH. Movies (Supplementary Movie 1–5) of the imaginal molt were recorded with a hand digital video camera (iVIS, Canon, Japan), by bringing late stage final instar nymphs back to a hotel room. All collecting was done correctly with the permission of MINAET-SINAC, Costa Rica, resolution numbers SINAC-SE-GASP-PI-R-122-2016, 056–2017-ACC-PI, M-P-SINAC-PNI-ACAT-047-2017.

### Micro-CT

In order to observe the adult helmet and developing nymphal helmet structure, we scanned treehoppers by using a micro–CT-scanner SkyScan 1172 (SkyScan NV, Belgium) following the manufacturer’s instructions. Sample preparation was performed as follows. Formalin fixed samples were dehydrated with 50/75/90/100% ethanol over 30 min in each step. Dehydrated samples were soaked in t-butanol and freeze-dried (FZ-2.5; Asahi Life Science, Japan). Dried samples were mounted on rubber putty to fix them during scanning. The X-ray source and resolution was 30–50 kV and 2.5 um/pixel, respectively. Suitable settings were used depending on the sample and purpose of the scan. Scanned primary shadow images were reconstructed into stacks of sections via SkyScan software NRecon (Version 1. 7. 1. 0) and then 3D volume-rendered images were constructed using SkyScan software CT Vox (Version 3. 3. 0). Movies and images of the constructed 3D volume-rendered images were also recorded by software function of CT Vox.

### Paraffin sections

Paraffin sections were prepared to observe the inner morphology of the helmet. Formalin fixed samples were transferred to 70% ethanol overnight and then dehydrated with 90, 95, and 100% ethanol, and finally cleared in xylene. Cleared samples were embedded into paraffin and sectioned (6 µm) with a microtome (RM2135, Leica, Germany). Sections were stained with hematoxylin and eosin, and captured with an all-in-one microscope (BZ-X, Keyence, Japan).

### Scanning electron microscopy (SEM)

Sample preparation was performed as follows. For observing the developing helmet inside the nymphal cuticle, we removed nymphal cuticles from formalin-fixed final instar nymphs with forceps under a binocular microscope. Dissected samples were then dehydrated with 50/75/90/100% ethanol for 30 min during each step. Samples were then soaked in t-butanol. The samples were dried with a freeze-dry system (FZ-2.5; Asahi Life Science, Japan) and coated with Au with DII-29010SCTR Smart Coater (JEOL Ltd., Tokyo, Japan). Prepared samples were observed with a scanning electron microscope JCM-6000plus (JEOL Ltd., Japan).

## Data Availability

The datasets used and/or analyzed during the current study are available from the corresponding author on reasonable request.
